# Comparison and Suggestions of Logistics Performance Index of Main Countries of Belt and Road Strategy Based on Bootstrap DEA Model

**DOI:** 10.1155/2022/2159578

**Published:** 2022-09-13

**Authors:** Wen-Tsao Pan, Bingqian Jiang, Yuting Wang, Yueyuan Cai, Xiaoxia Ji

**Affiliations:** ^1^School of Management, Guangzhou Huashang College, Guangzhou, China; ^2^School of Business, Guangdong University of Foreign Studies, Guangzhou, China

## Abstract

As an important economic sector, logistics is becoming more important, if not crucial, in economic growth. In our nation, the logistics industry is booming, and it's just getting better. However, in addition to focusing on the positive aspects of our country's logistics industry's development, we should also analyze and address the negative aspects of our country's logistics industry's development. The overall logistics pattern has not yet been formed, and there is an urgent need for systematic construction. The regional development is extremely unbalanced. By comparing the logistics performance indices of various Belt and Road countries, this research aims to examine the major elements influencing overall logistics performance. Second, we introduce the Moran index to explore the geographical association of the subdivision indicators of the logistics performance index using the spatial econometric model. The bootstrap DEA analysis method examines and ranks the countries' logistics performance indexes, determines our country's advantages and disadvantages in comparison to other Belt and Road countries, and executes specific improvement strategies that will enhance logistics and boost the overall growth of our country's logistics sector.

## 1. Introduction

As an important sector in the economic field, the logistics business plays an increasingly critical and even decisive role in economic development. In our nation, the logistics industry is booming, and it's just getting better. The logistics business in our nation has gradually developed since the founding of the People's Republic of China, and the amount of both supply and demand has expanded dramatically. However, in addition to focusing on the positive aspects of our country's logistics industry's development, we should also analyze and address the negative aspects of our country's logistics industry's development. The overall logistical plan has not yet been established, and systematic building is urgently required. There is a lack of specialization in the logistics industry, as well as few connected needs. Good international integration has been established; nevertheless, the level of logistics informatization is poor, and efficiency must be addressed immediately [1]. Logistics performance is frequently reflected by the Logistics Development Index as an important indicator of the level of logistics development (LPI). Low-performance logistics will increase trade costs, stifle the flow of goods, and weaken market competitiveness significantly. Customs clearance efficiency, logistics infrastructure quality, international transportation convenience, logistics service quality, ability to track products, and goods transit timeliness are all part of the logistics performance index system. The lower a country's trade expenses and the stronger its position in the global value chain, the higher its LPI score.

Tang Xiaoming et al., based on Tibet's geographical advantages and actual logistics development, analyzed the existing logistics development in Tibet Conditions, logistics nodes, and channels, and proposed the “five-in-one” Tibet logistics development strategy system framework [2]. Tao Zhang and Qiao Sen not only pointed out that national logistics performance has an impact on the “One Belt and One Road,” but also that national logistics performance has an impact on the “One Belt and One Road.” The route has a substantial positive impact on the amount of trade in the countries and regions that it passes through [3]. Wang Chao and others pointed out that, compared to foreign nations, notably industrialized countries in Europe and America, logistics performance evaluation technology is in its infancy. However, logistics performance analysis is beneficial to increasing a company's competitiveness and supporting its growth. As a result, future domestic study on logistics performance is crucial [4]. “Influence of Logistics Performance of the twenty-first Century Maritime Silk Road on China's Export of Mechanical and Electrical Products,” by Liu Zuankuo and colleagues, established an expanded trade gravity model and empirically analyzed the logistics performance of the twenty-first Century Maritime Silk Road. The essay makes specific policy recommendations based on the empirical findings [5].

The bulk of previous research has concentrated on a basic examination and analysis of the logistics efficiency index. The complete index of logistics efficiency index is rated and compared with a single analysis to assess its influence on the growth of our country's logistics industry and to define the development patterns and laws of the logistics industry. Wait. Existing research approaches include fuzzy comprehensive evaluation, multiple regression, entropy weight method, gray correlation analysis method, and others. No one has attempted a second in-depth analysis of the logistics development index using the bootstrap DEA technique.

This study intends to analyze the primary variables impacting overall logistics performance by comparing the logistics performance indices of different Belt and Road nations. Second, using the spatial econometric model, investigate the geographical correlation of the subdivision indicators of the logistics performance index by introducing the Moran index. The bootstrap DEA analysis method examines and ranks the logistics performance index of the countries involved, identifies our country's advantages and disadvantages in comparison to other Belt and Road countries, and implements targeted improvement measures that will promote the overall development of our country's logistics industry and improve logistics competitiveness.

## 2. Research Method

This study will adopt the following three research methods.

### 2.1. Visual Data Analysis

With the help of visual graphics software, we will show the lengthy data tables in the form of charts, such as line charts and bar charts, to make the data expression more visual and help to convey the expressed problems to the readers. Converting the original statistical tables into intuitive and visual charts is conducive to further comparison and analysis, as well as in-depth discovery. In terms of sample selection, we selected 22 countries as important representatives according to the world bank's logistics performance index and the distribution of countries along the “the Belt and Road”. The relevant logistics performance index and other data of 22 countries selected in this study are complete. The Central Asian economic belt includes Russia, Afghanistan, India, Pakistan, Iran, Turkey, Saudi Arabia, Iraq, Syria, and Jordan (the Asia Europe economic belt. Includes Germany, France, Britain, Italy, Ukraine, and Egypt). See Table 1 for the coverage and division of sections of the Silk Road Economic Belt.

### 2.2. Spatial Econometric Model

At the annual conference of the Netherlands Statistical Association, J. Paelinck first proposed spatial econometrics. As scientists continue to develop spatial autoregressive models, the spatial autoregressive model (SAR), the spatial error model (SEM), and the spatial Durbin model (SDM) are three classic spatial econometric models that are often used in research. The spatial autocorrelation model is one of them, and it seeks to determine whether a variable is connected in a geographical space area, as well as the degree of correlation. A typical statistic is the spatial autocorrelation coefficient. There are two types of autocorrelation: global space autocorrelation and local space autocorrelation. This approach is used to investigate the global and local spatial autocorrelation of China's territory in this study.

This study will realize the construction and analysis of the spatial econometrics model based on GeoDa software. As indicated by Moran's I, the global spatial autocorrelation mainly explores the degree of spatial reliance of each attribute variable over the whole area. Following data processing, the Moran scatter plot generated by the GeoDa program will be separated into four quadrants based on the attribute level of the area and the surrounding area, as well as the different types of the area space, namely high-high, high-low, low-low, and low-high. Scholars might use the divided four quadrants to see if a region has substantial spatial agglomeration characteristics.

Local spatial autocorrelation starts from the specific areas divided within the overall range and analyzes whether the space between attribute variables obeys the trend, that is, whether they are similar. First, we will use the GeoDa software to calculate the Moran index and use the results of the Moran index to get the change of attribute variables with the position. When the value is greater than 0, it is a positive correlation, and objects with similar attributes gather together, otherwise, objects with different attributes gather together. In addition, there is another possibility that when the Moran index approaches 0, it means that there is no spatial autocorrelation in the sample. Secondly, according to the results calculated by the single variable Moran index, the Lisa clustering map will be generated, and the local spatial autocorrelation will be visualized, which can further intuitively analyze the influencing factors.

### 2.3. Bootstrap DEA Model

Due to the restricted observation sample, it is difficult to avoid the issue of sample sensitivity and extreme value effect on the computed efficiency value since the DEA model has some of the benefits of different parameter estimation techniques (DMU). The efficiency value achieved with the DEA model is actually “relative efficiency.” In terms of absolute efficiency values, this estimate is biased and inconsistent. To overcome this flaw, Simar [6] et al. presented the Bootstrap-DEA method^19^, which can estimate the confidence interval, correct the bias of the DEA estimates, and establish the significance level^18^. The primary idea behind this method is to use the Bootstrap^20^ concept to sample the original samples repeatedly, create multiple Bootstrap sample data, collect many Bootstrap efficiency values, and construct confidence intervals using the empirical distribution of Bootstrap efficiency values. To increase the consistency of standard DEA estimators, use statistical inference. Overall, the DEA estimated efficiency value predicts the real efficiency value of the original sample, while the Bootstrap efficiency value is calculated by estimating and bias-correcting the DEA estimated efficiency value using a large number of simulated Bootstrap samples.

The method steps are as follows:

1. For each uses the traditional DEA method to calculate the efficiency value of the sample data
2. For the efficiency obtained in the first step, use the Bootstrap method to randomly sample *n* efficiency values where *b* represents the use of the b-the iteration of the Bootstrap method
3. Calculate the Bootstrap method to simulate the sample
4. Use the traditional DEA method to simulate each Bootstrap method sample and calculate the efficiency value again
5. Repeat steps 2–4 for a total of B times to generate the efficiency value

Three variables of Input (X), Output (Y), and DMUhbeen selected tedd for bootstrap DEA model analysis, and the DUM ranking and deviation correction under the combined action of two variables of *x* and *y* are discussed. Among them, DUM represents the name of the ranking unit being evaluated.

## 3. Empirical Analysis

### 3.1. Data Sources

The data used in this study are obtained from World Bank publications [7–11].

### 3.2. Variable Selection and Description

Table 2 shows the independent variable (INPUT) and dependent variable (OUTPUT) settings. Since the composite index is the upper-level indicator of other subindices according to the World Bank's official indicators, the composite index is used as the output index, while the other six subindices are used as the input index.

### 3.3. Research Methods and Data Computing

#### 3.3.1. Visual Chart Analysis

In 2007, 2010, 2012, 2014, 2016, and 2018, the World Bank published four logistics performance index reports. This post will use visual charts to compare the horizontal and vertical situations of the Belt and Road's logistics development, as well as the specific situation of our country's logistics development in the Belt and Road countries.

Due to a large amount of raw data, only the comprehensive logistics performance score is selected for visual analysis here.

**Figure 1:**
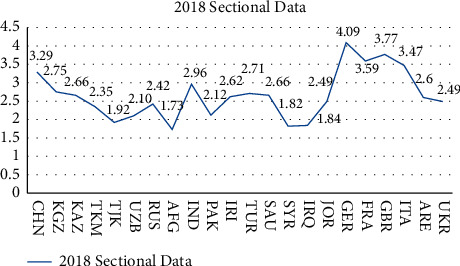
2018 logistics performance index of major belt and road countries: composite score (1 = very low to 5 = very high).

From the fluctuation trend of Figure 1, it can be seen that the comprehensive scores of the logistics performance of the major countries along the “Belt and Road” are quite different. The highest comprehensive score is Germany in the expansion area, which is as high as 4.20, while Afghanistan in the important area, whose comprehensive logistics performance score is only 1.73, is in a relatively inferior state, and the gap with other countries is obvious.

**Figure 2:**
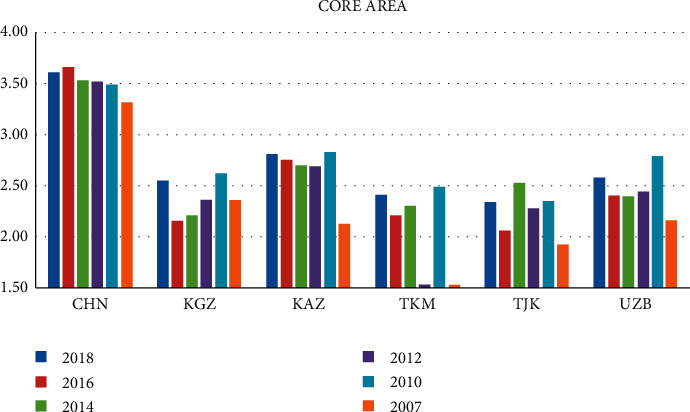
2007–2018 logistics performance index of core area: composite score (1 = very low to 5 = very high).

**Figure 3:**
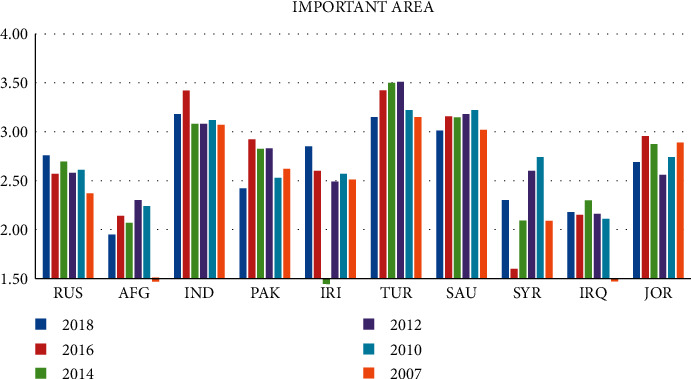
2007–2018 logistics performance index of important area: composite score (1 = very low to 5 = very high).

Figures 2 and 3 show the Logistics Performance Index for Core Area and Important Area from 2007 to 2018. On the whole, there is a large difference in the comprehensive scores of countries in important regions. Turkey has had a high comprehensive score in the past six years, followed by India and Saudi Arabia. However, compared with China, there is still a certain gap, which has not broken through 3.5. Afghanistan and Iraq have poor comprehensive scores of logistics performance, both of which are no more than 2.5. From the data, these two countries have low levels in the capacity and quality of logistics services and the quality of trade transportation-related infrastructure. They should strengthen exchanges with other countries and constantly improve own level in logistics services and trade and transportation.

**Figure 4:**
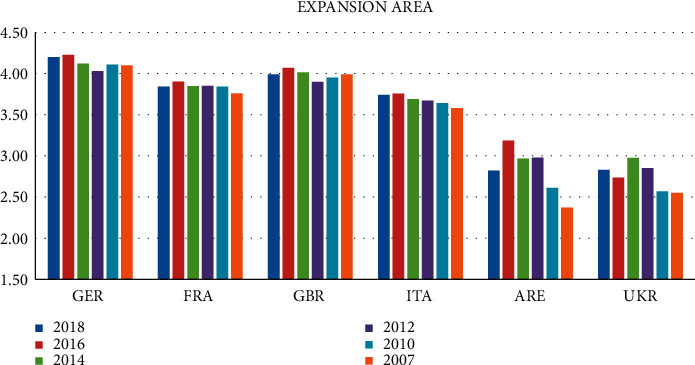
2007–2018 logistics performance index of expansion area: composite score (1 = very low to 5 = very high).

Of the countries in the expansion zone, Germany has the highest score, as shown in Figure 4. And its comprehensive score has stabilized at more than 4.0 from 2007 to 2018, followed by Britain, France, and Italy, with relatively high comprehensive scores. The Arab Republic of Egypt and Ukraine have the lowest comprehensive scores, and their logistics performance needs to be strengthened. It is not difficult to see that Germany has high scores in the ability and quality of logistics services, the ability to track and query goods, and the quality of trade and transportation-related structure. Although China is in a relatively stable state in all aspects, compared with Germany, China's logistics development level is still relatively backward. We should give full play to the comparative advantages of China's logistics industry and realize industrial upgrading according to the principles of complementary advantages, mutual benefit, and win-win results.

In general, our country's logistics development is at a medium level, and the logistics performance indexes of several established developed countries with a small proportion of expansion areas are all high. Among them, Germany ranks among the best in the past few years from 2007 to 2018. Followed. On the whole, the logistics development of the key countries of the “Belt and Road” has the characteristics of “the central part is poor, and the east and west are better”.

#### 3.3.2. Spatial Econometric Model

In this section, we will discuss the relationship between countries in the Belt and Road by using spatial econometrics and calculating the univariate Moran index by GeoDa software. Since GeoDa can only handle cross-sectional data, we only choose data for 2018. Table 2 shows the calculation results of each variable from *X*_1_–*X*_6_ with *Y*. It is evident from the results that the Moran's I of each variable is positive indicating that there is spatial autocorrelation between the independent variables and the dependent variable, and a positive correlation, indicating that objects with similar attributes are clustered together. Among them, the strongest correlation with the composite score is the ease of arranging competitively priced shipments.

**Figure 5:**
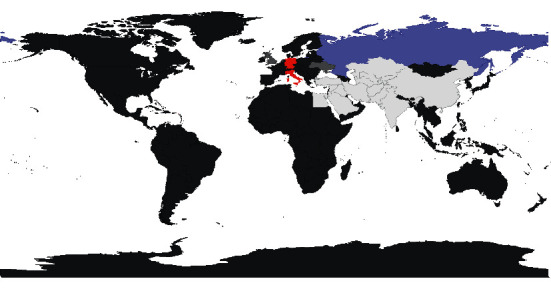
Moran cluster map between X1–X6 and Y.

Based on the calculation results in Table 3, LISA clustering maps were generated, as shown in Figure 5. Since the graphical results of the clustering maps generated by X_1_-X_6_ are almost the same, we have selected one of the six maps for display.

The LISA clustering map visualizes the local spatial autocorrelation and represents the impact of the “Belt and Road” on the countries along the “Belt and Road”. In the clustering map, the red area indicates High-High clustering; the blue area indicates Low-High clustering; the white area indicates insignificant spatial correlation; and the light gray area represents that the location has no neighbors among the 22 countries selected in this paper. The rest of the dark gray parts of the world map, which belong to the regions outside the research object of this paper, are not imported data.

In the figure, the regions of Germany and Italy, show High–High aggregation. This indicates that when these two regions have high LPI, the surrounding regions have correspondingly high scores. Russia, on the other hand, shows a Low-High aggregation, indicating that when Russia has a low logistics performance score, the surrounding regions have a high score instead. It can be seen that only individual countries show a more obvious spatial correlation in LPI changes, and the geographical connectivity among most countries along the Belt and Road has yet to be enhanced.

Then further spatial regression analysis was conducted in this paper by Spatial Error Model (SEM) and Spatial Lag Model (SLM), and the results are shown in Table 4. It is obvious that the numerical results of the two model runs are very similar, and there is an extremely strong explanatory power for both dependent variables (1 > 0.999978 > 0.999977). Also, the combination of LogL value (93.34 > 92.90), AIC value (0>−170.683>−171.803), SC value (−162.327>−164.492), and *p* value of LP test (0.5837 > 0.2773) shows that the Spatial Lag Model works better if the optimal spatial regression model is to be built. Since the focus of this paper is to explore the efficiency of logistics through MaxDEA, we will not expand on it here.

#### 3.3.3. Bootstrap DEA Model Analysis

The comprehensive score index (y1) and six subdivisions (Xi, *i* = 1,...,6) of the logistics performance of major nations along the Belt and Road in 2018 were extracted independently and entered into Max DEA for analysis.

In this article, the MAX DEA software is used to operate bootstrap DEA. First enter the original data, *X* includes 6 inputs x1, x2, x3, x4, x5, x6, and Y includes y1. Let DUM denote major economies. Select the envelope model, keep other default options, and select bootstrap for data analysis. The results are shown in Table 5.

The corrected values are re-ranked, and the results are as follows (Table 6).

From the analysis of the bootstrap DEA model, it can be seen that the first selected model, that is, the 2018 LPT report, comprehensively and completely describes and compares the logistics performance of major countries along the Belt and Road. The comprehensive ranking after the correction is more accurate and detailed than the original model, and the difference in comparison is more obvious, which is convenient for our further analysis. It can be seen that China's logistics performance is at a medium to a high level. China has performed well in terms of logistics capacity, international freight, timeliness, and cargo tracking, especially in infrastructure construction, but not outstanding in logistics performance rankings. This study speculates that the possible reasons are that, on the one hand, in terms of customs clearance, there are still problems such as cumbersome turnover procedures and low import and export efficiency; on the other hand, some logistics costs are low, such as low labor costs and high population density.

Compared with other countries, Germany has won the championship, followed by the United Kingdom, but the customs and infrastructure of the five Central Asian countries are relatively backward, and Uzbekistan's cargo tracking ability is medium, which needs to be further improved. Among them, Kazakhstan and other countries have a better delivery capacity, and Tajikistan performs better in international freight, ranking very high among the five Central Asian countries. At the same time, we can also see that the performance of several countries in the important regions of the Central Asian Economic Belt is quite different. In the context of “One Belt, One Road”, in line with the principle of complementary advantages, mutual benefit, and win-win, countries should conduct in-depth exchanges to jointly promote the improvement of logistics levels.

## 4. Conclusions

By comparing the logistics performance indexes of various countries in the Belt and Road, this paper explores the main factors affecting the comprehensive performance of logistics, introduces the Moran index, and uses the spatial econometric model to explore the geographical relationship of the subdivision indicators of the logistics performance index. The country's logistics performance index is compared.

From the visualization analysis and bootstrap DEA model analysis, it can be seen that China's logistics is at a medium level, and its scores are mainly in the efficiency of customs clearance procedures and the quality of trade and transportation-related infrastructure. So far, there are still large logistics vacancies along the Belt and Road. On the Silk Road Economic Belt, European countries have relatively high incomes and logistics performance indices, whereas other countries and regions, such as the five Central Asian countries, have lower national logistics performance indices, with their highest levels of logistics performance at the bottom. The data shows that in 2014, compared with Afghanistan, the best-performing country, Afghanistan was 2.05 points worse than Germany, and Afghanistan ranked 158 in that year. In 2012, the logistics performance of various countries was 141, and the corresponding score was 141.1.87. China, Turkey, and India in the economic belt are middle- and upper-income countries, and their logistics performance is correspondingly high. This “logistics vacancy” is not conducive to the overall development of the economic belt.

However, the relationship between regional income level and logistics performance is less well understood than the relationship between physical location and logistical performance. Overall, countries with higher incomes also have higher logistics performance, but looking at the relative performance of incomes aside from absolute incomes, there is no evident link between earnings and performance improvement. Taking our country as an example, in 2014, our country's per capita income ranked 94th in the world, at US$7,476, but our country's global ranking on the logistics performance index was 28, which is an example of lower-income and higher logistics performance ranking. Moreover, for countries with extremely uneven regional distribution, such as our neighbor Russia, there are large intra-country differences, and the LPI score cannot well reflect the country's logistics development level.

Here, this study makes the following recommendations:

1. It is necessary to improve the customs clearance mechanism and customs clearance coordination mechanism of the countries along the route, establish the “Belt and Road” free trade zone, improve customs efficiency, and reduce the difference in customs clearance efficiency. Accelerate the improvement of the customs clearance coordination mechanism between China and other countries along the Belt and Road, solve the problems of low customs clearance efficiency and low timeliness of logistics services, make a trade in services and goods more convenient, and promote the facilitation and efficiency of economic and trade exchanges between countries. At the same time, it is necessary to strengthen the infrastructure construction cooperation with the countries along the “Belt and Road” to communicate with each other, speed up the improvement of logistics infrastructure, maintain and upgrade existing equipment, and improve the operating efficiency of existing facilities, increase the net throughput of ports, and reduce construction development of new technical equipment.
2. Strengthen the development of regional linkage of logistics system among Belt and Road countries. The above spatial econometric analysis results show that the main countries of the Belt and Road have weak spatial correlations, while the Belt and Road aim to carry out wider, higher-level, and deeper regional cooperation, and advocates the interconnection of countries. Territorial connectivity is essential. As a logistics industry with prominent geographical elements, it plays an indispensable role in enhancing spatial correlation. In the future, China should strengthen trade links with countries along the route with high levels of economic development, a high degree of openness, and strong radiation capabilities. It is conducive to driving the liquidity of the entire “Belt and Road” trade, improving the level of logistics, and enhancing the regional connectivity between countries.
3. Improve the efficiency of infrastructure development and customs passage. The five Central Asian countries are the only way for the Silk Road in terms of geographical conditions, but it is precise because of their disadvantageous geographical conditions that their geographical conditions are poor and their resource distribution is extremely uneven, resulting in its Economic development lagging behind other coastal countries and regions with rich products and superior natural conditions. In 2014, only Kazakhstan's logistics performance ranked among the top 100 in the world, ranking 88th. Only in 2010 did the five Central Asian countries have their best year in the logistics performance index, and they all joined the world's top 100 for the first and only time. The five Central Asian countries are relatively backward in all aspects of logistics. Infrastructure construction and customs clearance efficiency must both be enhanced, in the end [6].
4. Develop green ecological logistics. “Lucid lakes and beautiful mountains are priceless assets,” says the logistics business, which also requires green growth to have a low environmental impact. Ecological issues are not just national or regional issue; they are also a global issue. Many nations will respond positively to the request to develop a green logistics ecological chain, as worldwide attention to environmental issues has increased in recent years. Active participation is required. To limit the negative effect on the environment, we must implement ecological civilization in all parts of logistics, from the procurement of raw materials to delivery to clients, and aim to reduce resource consumption, carbon emissions, and recycling as much as feasible [12]. The Green Silk Road's economic logistics are collaboratively constructed and shared by all countries.

## Figures and Tables

**Table 1:** The coverage and division of sections of the Silk Road Economic Belt.

Arrangement	Region	Major economies	Country abbreviation
Core area	Central Asian economic Belt (five Central Asian countries)	China	CHN
		Kyrgyzstan	KGZ
		Kazakhstan	KAZ
		Turkmenistan	TKM
		Tajikistan	TJK
		Uzbekistan	UZB

Important area	Central Asia Economic Belt (Central Asia, West Asia, Russia, India, Pakistan)	The Russian Federation	RUS
		Afghanistan	AFG
		India	IND
		Pakistan	PAK
		Islamic Republic of Iran	IRI
		Turkey	TUR
		Saudi Arabia	SAU
		Syrian Arab Republic	SYR
		Iraq	IRQ
		Jordan	JOR

Expansion area	Asia Europe Economic Belt (Central Asia, Europe and North Africa)	Germany	GER
		France	FRA
		The U.K.	GBR
		Italy	ITA
		Arab Republic of Egypt	ARE
		Ukraine	UKR

**Table 2:** INPUT and OUTPUT introduction.

OUTPUT (Y)	INPUT (X)
Y_1_: composite index	X_1_: how often do the goods arrive at the consignee within the scheduled or expected time
	X_2_: quality of trade and transport-related infrastructure
	X_3_: efficiency of customs clearance procedures
	X_4_: ability to track and inquire about goods
	X_5_: ease of arranging competitively priced shipments
	X_6_: ability and quality of logistics services

**Table 3:** Moran's I calculation results.

	X1	X2	X3	X4	X5	X6
Moran's I	0.486	0.495	0.510	0.530	0.536	0.487

**Table 4:** Results of spatial regression model analysis.

	R-squared	Log-likelihood	Akaike info criterion	Schwarz criterion	Likelihood ratio test prob
SEM	0.999977	92.901735	−171.803	−164.492	0.58370
SLM	0.999978	93.3417	−170.683	−162.327	0.27730

**Table 5:** Results raw data.

No.	DMU	Score (original)	Bias	Mean	Median	Sd	CI_LowerBound	CI_UpperBound
1	Afghanistan	1	0.0018889	1.0018889	1.0009291	0.0026905	1.0001232	1.0114327
2	Egypt	0.9928662	0.0008557	0.9937219	0.9935008	0.0007915	0.9929146	0.996882
3	Pakistan	1	0.0019365	1.0019365	1.0009292	0.0027918	1.0000848	1.0107582
4	Germany	1	0.0021274	1.0021274	1.0010236	0.002828	1.000059	1.0115292
5	Russia	1	0.0013026	1.0013026	1.0009146	0.0014179	1.0000634	1.0074079
6	France	1	0.001225	1.001225	1.0008382	0.0011593	1.0000513	1.0052738
7	Kazakhstan	1	0.0013736	1.0013736	1.0009893	0.0013802	1.0000678	1.0068713
8	Kyrgyzstan	1	0.0020506	1.0020506	1.000973	0.00278	1.0000731	1.0115743
9	Saudi	1	0.0018772	1.0018772	1.0009005	0.0026984	1.0000672	1.0111135
10	Tajikistan	1	0.0016653	1.0016653	1.0008854	0.0024274	1.0000731	1.0110763
11	Turkey	1	0.0013324	1.0013324	1.0009115	0.0012414	1.0000479	1.0057658
12	Turkmenistan	1	0.0020379	1.0020379	1.0009489	0.0028231	1.0000851	1.0110627
13	Ukraine	1	0.0021894	1.0021894	1.0009496	0.0029749	1.000069	1.0120212
14	Uzbekistan	1	0.00194	1.00194	1.0009283	0.0025734	1.0000439	1.0100235
15	Syria	1	0.0022273	1.0022273	1.0009279	0.0029162	1.000093	1.0103861
16	Iraq	1	0.0019241	1.0019241	1.0009189	0.0026837	1.0000542	1.0106874
17	Iran	0.9894613	0.0008688	0.9903301	0.9901487	0.0006542	0.9895422	0.9930757
18	Italy	0.9990447	0.0008299	0.9998746	0.9997653	0.0005505	0.999129	1.0025317
19	India	0.9914746	0.0009293	0.9924039	0.9921703	0.0007987	0.9915093	0.9961749
20	The U.K.	1	0.0013702	1.0013702	1.0009941	0.0012389	1.0001018	1.0058977
21	Jordan	1	0.0020418	1.0020418	1.0009684	0.0028026	1.0000804	1.0118942
22	China	1	0.0016956	1.0016956	1.0007966	0.0022515	1.0000791	1.0083734

**Table 6:** Ranking results.

Rank	DMU	Median
1	Germany	1.001023584
2	U.K.	1.000994064
3	France	1.000989313
4	Italy	1.000973002
5	China	1.000968445
6	India	1.00094961
7	Turkey	1.00094893
8	Russia	1.000929174
9	Kazakhstan	1.00092911
10	Kyrgyzstan	1000928327
11	Jordan	1.000927938
12	Ukraine	1.000918908
13	Turkmenistan	1.000914579
14	Pakistan	1.000911534
15	Afghanistan	1.000900459
16	Uzbekistan	1.000885444
17	Syria	1.000838196
18	Iraq	1.000796621
19	Saudi	0.999765322
20	Tajikistan	0.993500798
21	Egypt	0.992170308
22	Iran	0.990148732

## Data Availability

The data used to support the findings of this study are available from the corresponding author upon request.
